# Water Soluble Antioxidative Crude Polysaccharide From *Russula senecis* Elicits TLR Modulated NF-κB Signaling Pathway and Pro-inflammatory Response in Murine Macrophages

**DOI:** 10.3389/fphar.2018.00985

**Published:** 2018-08-28

**Authors:** Somanjana Khatua, Krishnendu Acharya

**Affiliations:** Molecular and Applied Mycology and Plant Pathology Laboratory, Department of Botany, Centre of Advanced Study, University of Calcutta, Kolkata, India

**Keywords:** antioxidant activity, ethnic food, immuno-stimulatory ingredients, molecular conformation, RAW 264.7 cells, mushroom β-glucan

## Abstract

*Russula senecis* has recently been reported as a new addition to macrofungal flora of West Bengal. Besides, it also emerged as a seasonal health promoting nutrient to local ethnic people and enlisted for the first time as tribal food in our previous publication. In this context, the present work was designed to establish such usefulness scientifically and to meet the aim, crude polysaccharide, Rusenan, was prepared using conventional heated water reflux. Initially, the polymers were characterized to determine chemical composition and for that spectrophotometry along with Fourier-transform infrared spectroscopy (FT-IR), high-performance thin-layer chromatography (HPTLC), and gas chromatography-mass spectrometry (GC-MS) were performed. Analysis indicated that Rusenan was consisted mainly of carbohydrate conjugated with trace amount of protein. Furthermore, glucose was detected as the major monosaccharide (mainly in β-type glycosidic linkage) while other monomers were presented in the order of galactose > mannose > xylose > rhamnose. Conversely, antioxidant potential was determined following eight *in vitro* systems where the fraction evidenced strong superoxide, hydroxyl, 2,2-diphenyl-1-picrylhydrazyl (DPPH) and 2,2′-azino-bis(3-ethylbenzothiazoline-6-sulphonic acid) (ABTS) radical scavenging activity, high affinity to Fe^2+^ as well as instant ability to donate electron with EC_50_ values ranging from 80 to 3885 μg/ml concentration. In addition, effect on murine macrophages was also investigated where the polysaccharide treatment increased cell proliferation, phagocytic activity, filopodia or lamellipodia formation, nitric oxide (NO) production and reactive oxygen species (ROS) synthesis. Thereafter, through reverse transcriptase polymerase chain reaction (RT-PCR) analysis, significant increase in the expression of Toll like receptor (TLR)-4, TLR-2 and nuclear factor kappa B (NF-κB) was observed; as a result alleviated level of cyclooxygenase (COX)-2, inducible nitric oxide synthase (iNOS), tumor necrosis factor (TNF)-α, IκB-α, and interferon (IFN)-γ were also noticed explaining definite immune-stimulatory activity of the fraction. Thus, overall finding suggests that *R. senecis* can be considered as a functional food and may be used in preparation of dietary supplement to enhance general health.

## Introduction

Botanical polysaccharides exhibit diverse therapeutic properties and the effect is thought to be related to modulation of innate immunity more precisely macrophage function (Venkatalakshmi et al., 2016). Macrophages are long-lived cellular effector of innate immunity and the major cell type involved in stimulating adaptive immune response (Wang et al., 2013). They are the first cells that come in contact with invader microorganisms and are essential for their elimination (Li et al., 2015). The clearance process starts in part by TLRs of the monocytes that bind to protein, lectin, lipoprotein and polysaccharide of pathogens. Subsequently, TLRs trigger downstream cascade that eventually activate transcription factor, NF-κB which sets off a series of reactions producing pro-inflammatory cytokines and chemokines in a sequential manner. The behavior induces both T and B lymphocytes initiating adaptive immune response (Duque and Descoteaux, 2014). Hence, the agents that has ability to augment macrophage function is highly important to improve overall immunity especially to those suffering from cancer, AIDS and auto-immune diseases (Lee et al., 2013). Thus, macrophages are considered as a perfect model for scientific evaluation of immune-stimulatory agent. In that note, being the most abundant bioactive polymers, polysaccharides provide a unique opportunity for discovery of novel therapeutics (Venkatalakshmi et al., 2016).

In addition, many studies also reveal that polysaccharides play an important role as free radical scavengers and antioxidants for prevention of oxidative damage (Khatua et al., 2013). Consequently, oxidant mediated tissue injury is a hazard to the immune system being sensitive to free radical induced stress. Immune cells rely heavily on membrane bound receptors to work effectively via cell–cell communication. However, these cellular membranes rich in polyunsaturated fatty acids are the most susceptible to free radicals and the attack can lead to alteration in intracellular signaling (Hughes, 1999). At this point, antioxidants play a fundamental role in maintaining optimal health as it represents the first line of defense against oxidative damage by scavenging radicals (Khatua et al., 2013). By contrast, intense suppression of immune functions might be observed due to insufficient status of dietary antioxidants that increases risk of infection (Puertollano et al., 2011).

In this scenario, mushroom polysaccharides have been appreciated in human diet and traditional medicine especially in Far Asia. However, their popularity has recently been increased due to growing awareness of their therapeutic properties supported by scientific research (Giavasis, 2014). Several such biopolymers especially β-glucan are ascribed miraculous power of conferring longevity and strengthening immune system. Studies have revealed that these immunomodulators could proliferate and activate innate immune components including macrophages (Akramienė et al., 2007). Besides, these naturally occurring glucose polymers have also been approved to scavenge free radicals and reduce components (Khatua et al., 2013). Thus, edible mushrooms and their polysaccharides are recognized at present as functional food ingredients and considered to improve general health via immune-stimulatory as well as antioxidant potential (Akramienė et al., 2007; Vlatka et al., 2010).

Despite the effects, information on chemical constituent and potent health benefit of several macrofungi is scarce, even today. One such less explored taxon is *Russula senecis* that has recently been documented as a new member to macrofungal flora of West Bengal, India. Interestingly, the specimen also emerged as a seasonal health promoting food among local indigenous people indicating safety for human consumption (Khatua et al., 2015). Recently, medicinal activity of polysaccharide from the mushroom has been established in our previous study, although 10% NaOH solution was used as an extractant solvent (Khatua and Acharya, 2017). Nevertheless, studies need to be conducted concentrating on conventional hydrothermal process as mushrooms are traditionally ingested by cooking in water. Therefore, the present work was designed to provide insight into chemical composition of polysaccharides from *R. senecis* prepared by heated water reflux. Further, the formulation was evaluated for putative therapeutic activities namely antioxidant and immune-enhancing effects to predict usefulness of the folk mushroom.

## Materials and Methods

### Chemicals

2-Deoxy-D-ribose, ferric chloride, hydrogen peroxide, L-methionine, thiobarbituric acid, nitroblue tetrazolium (NBT), ferrozine, riboflavin, trichloroacetic acid, potassium ferricyanide, DPPH, ABTS, trifluoroacetic acid, sodium borohydride, sodium persulfate, ammonium molybdate, toluene, pyridine, dichloromethane, ascorbic acid, ethylene diaminetetraacetic acid (EDTA), butylated hydroxyanisole (BHA), gallic acid, bovine serum albumin (BSA), lipopolysaccharide (LPS) (extraction from *Escherichia coli* 026:B6) and monosaccharides were procured from Sigma Chemical Co. (St. Louis, MO, United States). Toluene, dichloromethane, chloroform were of HPLC grade and monosaccharides were of extra pure form. A mushroom β glucan kit from Megazyme Institute Wicklow, Ireland was used. Dulbecco’s Modified Eagles Medium (DMEM), sulfanilamide, neutral red, naphthylethylenediamine dihydrochloride, Congo red, phosphoric acid, 4′,6–diamidino–2–phenylindole (DAPI), 2′,7′–dichlorofluorescin diacetate (DCFDA), were purchased from Himedia, Mumbai, India. Fetal bovine serum (FBS) and water soluble tetrazolium (WST) were purchased from Takara Bio Inc, Japan and Invitrogen, Carlsbad, CA, United States, respectively. Amphotericin B and PenStrep were used from MP Biomedicals, Santa Ana, CA, United States. A two-step reverse transcriptase-PCR (RT-PCR) kit from MP Biomedicals, Mumbai, India was used for cDNA synthesis from isolated RNA.

### Collection and Authentication of Basidiocarps

Fruit bodies of *R. senecis* were collected from natural habitat of West Bengal in the month of July. Identity of the basidiome was confirmed based on macro and micro-morphological studies accompanied by phylogenetic analysis as described in our previous publication (Khatua et al., 2015). Representative voucher specimen (Accession no: CUH AM102) was deposited at herbarium in the same department of University of Calcutta.

### Extraction of Crude Polysaccharide (Rusenan)

Dried and powdered fruit bodies were steeped with ethanol (approximately 10 volume) overnight to eliminate the alcohol soluble components and filtered residue was re-extracted with ethanol. The air dried filtrate was suspended and refluxed with distilled water at boiling condition for 7 h. Subsequently, the extract was filtered through nylon cloth and lyophilized to concentrate. Four volumes of absolute ethanol was added for harvesting polysaccharide and incubated overnight at 4°C. After centrifugation (11,000 rpm for 10 min at 4°C), pellets were dissolved in water repeatedly to obtain crude polysaccharide fraction that was highly soluble in water. Alcohol precipitated pellets were recovered by centrifugation followed by washing with ethanol and acetone. The crude polysaccharide fraction was designated as Rusenan and kept in amber containers under dry condition until analyzed (Khatua et al., 2017a).

### Structural Characterization of Rusenan

Total sugar content was measured by phenol sulfuric acid method using glucose as standard and results were expressed as gm of glucose equivalents/100 g of dry polysaccharide. α-glucan, β-glucan, and total glucan were estimated using mushroom and yeast β -glucan assay kit as per the manual. Protein concentration was determined using the method described by Bradford reagent and quantity was expressed as gm of BSA equivalents/100 g of dry polysaccharide. Moreover, Rusenan was examined using FT-IR (PerkinElmer Inc., Spectrum 100, Waltham, MA, United States) in frequency range of 400–4000 cm^-1^. Further, the molecular composition was analyzed by HPTLC as well as GC-MS (Khatua and Acharya, 2016).

### Evaluation of Antioxidant Activities of Rusenan *in vitro*

The assay of total antioxidant capacity was carried out and activity was expressed as number of equivalent of BHA (Prieto et al., 1999). Besides, superoxide radical scavenging activity of Rusenan (200–600 μg/ml) was evaluated based on riboflavin-light-NBT system using 96 well plate and absorbance was measured by microplate reader (Bio-Rad iMark^TM^ Microplate Reader, United States) (Khatua et al., 2017b). The method described by Halliwell et al. (1987) was followed for determination of hydroxyl radical (OH^.^) scavenging activity. The radicals were generated by Fenton’s reaction in presence of variable concentrations (200–500 μg/ml) of Rusenan and absorbance was measured at 535 nm. In addition, the antioxidant activity was also evaluated using DPPH radical adopting microtiter plate. DMSO solution of the radical (0.1 mM) was evaluated against various concentrations of crude polysaccharide (500–1500 μg/ml) and absorbance was detected at 517 nm. The assay of ABTS radical scavenging activity was performed as well using a range of fraction (100–1000 μg/ml) (Khatua et al., 2017b). Further, the potential of Rusenan (100–500 μg/ml) was also evaluated by β-carotene linoleate model system (Dapkevicius et al., 1998). Moreover, the ability of investigated extract to chelate ferrous ion was determined using different concentrations of Rusenan (50–200 μg/ml). A modified method of reducing power was considered also. Variable doses of the extract (2000–4000 μg/ml) were mixed in 60 μl reaction mixture and the absorbance was measured at 750 nm (Khatua et al., 2017b). BHA was considered as standard in superoxide, hydroxyl and DPPH radical scavenging assays, reducing power technique along with inhibition of β-carotene bleaching assay; while EDTA was adopted as a positive control in chelating ability of ferrous ion method. The sample concentration providing 50% of antioxidant activity or 0.5 of absorbance in reducing power assay were calculated from graphs of antioxidant activity percentages and regarded as EC_50_ value.

### Determination of Immune-Stimulatory Potential of Rusenan

#### Effect on Macrophage Cell Proliferation, Phagocytosis, NO Production, ROS Synthesis and Morphology

RAW 264.7 murine macrophages were purchased from NCCS, Pune, India and maintained in DMEM supplemented with 10% (v/v) FBS, 0.5% (v/v) PenStrep (5,000 IU/ml penicillin and 5 mg/ml streptomycin) and 0.25% (v/v) amphotericin B (250 μg/ml). Initially, about 3000 cells were seeded in 96-well plate overnight, Rusenan at variable doses (50, 100, and 200 μg/ml) was added to 200 μl reaction mixture and incubated for different time intervals. Subsequently, 20 μl WST reagent was added and absorbance was measured at 450 nm. To investigate effect on pinocytic activity, reaction mixture after definite period of incubation was replaced with 100 μl DMEM containing 0.07% (w/v) neutral red followed by washing the cells. Then 100 μl of cell lysate reagent (ethanol:0.01% acetic acid = 1:1) was added and after 2 h optical density was measured at 575 nm. Further, NO release was estimated by adding 100 μl of Griess reagent in 100 μl reactant and absorbance was noted at 545 nm. Besides, change in cellular morphology was observed after 24 h treatment under inverted microscope (FLoid^®^ Cell Imaging Station, Life Technologies, Carlsbad, CA, United States) using a dye, DAPI. In addition, consequence on intracellular ROS production was measured with flow cytometry (BD Bioscience, San Jose, CA, United States) using DCFDA and analyzed by BD CellQuest Pro software. In all experimental sets, LPS at 5 μg/ml concentration was used as a positive control (Khatua and Acharya, 2017; Khatua et al., 2017a).

#### Measurement of Gene Expression by RT-PCR Analysis

RAW cells (6 × 10^5^ cells/well) were treated with various concentrations of Rusenan and allowed for incubation of 24 h. Further, RNA was extracted using TRIzol reagent and reverse transcription was performed with 1 μg of total RNA using RT-and GO Mastermix according to the manufacturer’s instructions. PCR (Thermo Fisher Scientific Incorporated, United States) was performed using primers for TLR-4, TLR-2, NF-κB, IκB-α, COX-2, iNOS, TNF-α, and IFN-γ genes at different annealing temperature with β-actin as internal control (**Table 1**). For each gel, ImageJ software was applied for quantitative measurement of band intensity.

**Table 1:** The primer sequences used to determine immune-stimulatory effect.

Gene of interest	Primer sequence	Tm (°C)	Reference
TLR–4	F: 5′CAGCTTCAATGGTGCCATCA3′	54	Fu et al., 2006
	R: 5′CTGCAATCAAGAGTGCTGAG3′		
TLR–2	F: 5′CACCACTGCCCGTAGATGAAG3′	57	Liu et al., 2017
	R: 5′AGGGTACAGTCGTCGAACTCT3′		
NF–κB	F: 5′AGAAGGCTGGGGTCAATCTT3′	51	Sun et al., 2017
	R: 5′CTCAGGCTTTGTAGCCAAGG3′		
IFN-γ	F: 5′CCTCAAACTTGGCAATACTCA3′	54	
	R: 5′CTCAAGTGGCATAGATGTGGA3′		
Iκ–Bα	F: 5′CTTGGTGACTTTGGGTGCTGAT3′	57	Terra et al., 2007
	R: 5′GCGAAACCAGGTCAGGATTC3′		
iNOS	F: 5′GAGCGAGTTGTGGATTGTC3′	55	Khatua et al., 2017a
	R: 5′GGGAGGAGCTGATGGAGT3′		
COX–2	F: 5′CCCCCACAGTCAAAGACACT3′	57	
	R: 5′GAGTCCATGTTCCAGGAGGA3′		
TNF–α	F: 5′ATGAGCACAGAAAGCATGATC3′	56	
	R: 5′TACAGGCTTGTCACTCGAATT3′		
β-Actin	F: 5′GCTGTCCCTGTATGCCTCT3′	55	
	R: 5′TTGATGTCACGCACGATTT3′		


### Statistical Analysis

IBM SPSS Statistics for Windows, version 23.0. (IBM Corp., Armonk, NY, United States) was used as statistical analysis software. Experiments were performed at least in triplicate and results are presented as mean ± standard deviation (SD). One-way analysis of variance (ANOVA) followed by Tukey’s *post hoc* test was employed to assess significant differences (*p* < 0.05).

## Results and Discussion

### Determination of Extractive Yield and Major Constituents

The crude polysaccharide, Rusenan, was prepared from *R. senecis* after hot water reflux and ethanol precipitation that appeared as whitish powder and highly soluble in water. The extraction yield was determined as 4.42 ± 0.26% by dry weight that exceeded leaching efficacy of *Russula virescens* (Sun et al., 2010), *Russula alatoreticula* (Khatua et al., 2017a), *Macrocybe gigantea* (Khatua and Acharya, 2016) and *Termitomyces eurhizus* (Chatterjee et al., 2013). However, the recovery percentage of Rusenan was found to be 2.2 times lower than that of the fraction isolated by alkaline solvent from *R. senecis* (Khatua and Acharya, 2017).

Subsequent characterization clearly showed that the prepared fraction was polysaccharide rich as total carbohydrate content was 39.79 ± 4.52 g/100g dry polysaccharide and quantity of protein was 4.01 ± 0.06 g/100g dry polysaccharide. Further, absolute predominance of β-glucan in Rusenan was detected as total glucan, β-glucan and α-glucan content were 15.63 ± 2.49, 12.11 ± 0.28, and 3.52 ± 2.51 g/100g dry polysaccharide, respectively. Comparative study with previous literature indicated that *R. senecis* might be consisted of more carbohydrate than that of *R. alatoreticula* (Khatua et al., 2017a), *T. eurhizus* (Chatterjee et al., 2013) and *Schizophyllum commune* (Klaus et al., 2011). Besides, the content of β-glucan as well as α-glucan were found to be in higher amount than that of *Pleurotus florida* (Saha et al., 2013) while the extent of protein was better than the hot water extract from *Ganoderma lucidum* and *Agaricus bisporus* (Kozarski et al., 2011).

### FT-IR Spectroscopic Analysis

**FIGURE 1:**
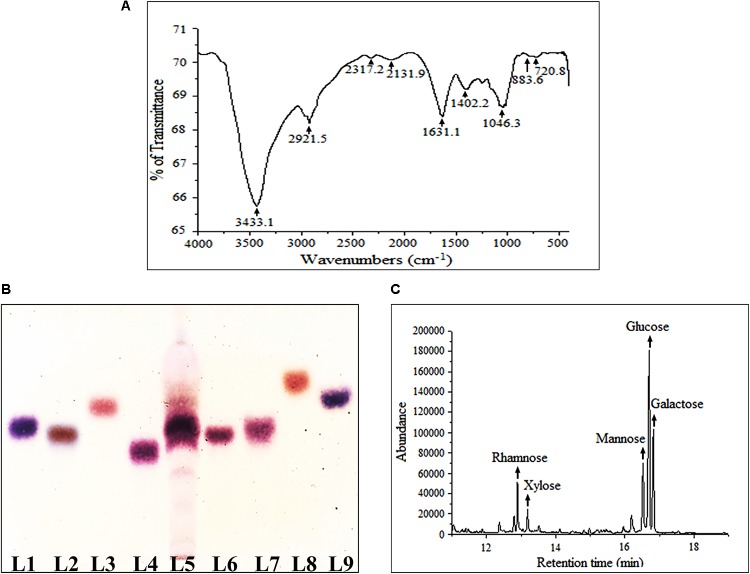
Structural and molecular characterization of crude polysaccharide, Rusenan, isolated from *Russula senecis.* **(A)** FT-IR spectrum **(B)** Identification of monosaccharides in hydrolyzed polysaccharides by HPTLC, Lanes: 1: L-arabinose, 2: D-fructose, 3: D-fucose, 4: D-galactose, 5: Rusenan, 6: D-glucose, 7: D-mannose, 8: D-rhamnose, 9: D-xylose. **(C)** GC-MS chromatogram of derivatized Rusenan (Retention time of D-xylose: 12.8 min, D-rhamnose: 13.2 min, D-mannose: 16.6 min, D-glucose: 16.7 min, D-galactose: 16.8 min).

FT-IR was performed herein to identify functional groups present in Rusenan (**Figure 1A**). As described in previous literature, presence of strong and broad absorption band at 3433.1 cm^-1^ was due to -OH stretching indicates strong inter- and intramolecular connection between polysaccharide chains. Peak at 2921.5 cm^-1^ could be attributed to stretching vibration of the C–H bond in sugar ring. The signal at 1631.1 cm^-1^ corresponded to carbonyl group of carboxylic acid group. Absorption at 1402.2 cm^-1^ represented OH group deformation vibration in plane. The peak at 1243.3 cm^-1^ indicated presence of o-acetyl groups. Band at 1046.3 cm^-1^ was due to C-O-C unsymmetrical stretching and confirmed presence of mannopyranose compound. Further, existence of β-glycosidic bonds was indicated by characteristic absorbance at 883.6 cm^-1^. Peak at 720.8 cm^-1^ validated presence of mannose in Rusenan (Synytsya et al., 2009; Klaus et al., 2011; Bayar et al., 2016; Preethi and Mary, 2016; Shi et al., 2016). Overall, the spectrum presented high absorbance at wave numbers of 3400 and 1200–800 cm^-1^ that were characteristic of polysaccharide. Based on the outcome, it could be interpreted that Rusenan was mainly consisted of sugar units in β-configuration along with small amount of lipid and protein.

### Determination of Monosaccharide Composition

The most popular method to determine core structure of polysaccharide is TLC followed by orcinol-H_2_SO_4_ spray as they execute specific color after reacting differently with hexose and pentose sugars (Ruthes et al., 2015). In the present study, types of monosaccharides in Rusenan was preliminarily analyzed by HPTLC and the chromatogram was found to be consisted of four monosaccharides like galactose, glucose, mannose, and xylose (**Figure 1B**). The result was further authenticated by GC-MS and for that the polar carbohydrates were transformed to non-polar components through hydrolysis, reduction as well as acetylation. The fingerprint profile was detected as quite similar to previous result except rhamnose which was not detected in TLC chromatogram as it was present in trace. Thus Rusenan was found to be composed of total five types of monomers such as xylose, rhamnose, mannose, glucose, and galactose presented in the molar ratio of 11.86: 5.71: 16.18: 42.24: 24.01 (**Figure 1C**). Interestingly, the alkaline extracted polysaccharide from *R. senecis* was found to be composed of only four units like xylose, rhamnose, mannose, and glucose where all monosaccharides were in insignificant amount except glucose (Khatua and Acharya, 2017). Thus the observation might indicated that hot water process facilitates extraction of different types of monomers in higher extent than other solvent types.

### Evaluation of Antioxidant Activities of Rusenan *in vitro*

**FIGURE 2:**
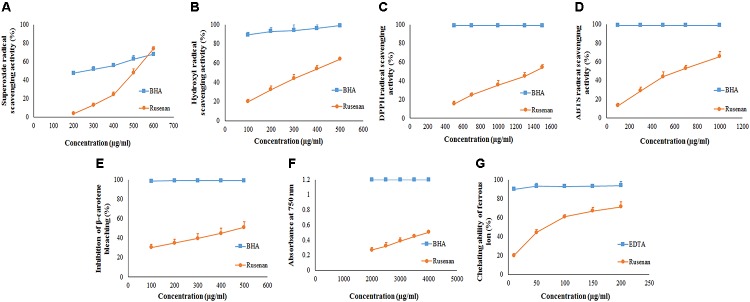
Antioxidant activity of crude polysaccharide, Rusenan, prepared from *Russula senecis.* **(A)** Superoxide radical scavenging activity. **(B)** Hydroxyl radical scavenging activity. **(C)** DPPH radical scavenging activity. **(D)** ABTS radical scavenging activity. **(E)** β-carotene bleaching assay. **(F)** Reducing power **(G)** Chelating ability of ferrous ion (Results represent mean ± SD of at least three independent experiments).

To evaluate antioxidant ability of Rusenan total eight *in vitro* methods were executed herein and the activities have been summarized in **Table 2**. Initially, to estimate radical scavenging activity four techniques were adopted like superoxide, hydroxyl, DPPH and ABTS radicals scavenging assays. Results demonstrated that Rusenan exhibited strong concentration-dependent quenching effects of the superoxide radicals. At the concentration of 200 and 400 μg/ml, the extract was able to inhibit 3.8 and 24.33% radicals, respectively, which reached to the level of 74% at only 600 μg/ml concentration (**Figure 2A**). Besides, a kinetic approach was adopted to assess OH^.^ scavenging properties of Rusenan. Outcome of the method has been presented in **Figure 2B** which showed strong concentration dependent inhibition of the radicals at relatively low concentrations. The scavenging activities were found to be 32.46, 44.29, and 64.17% at the concentrations of 200, 300, and 500 μg/ml, respectively. Moreover, in order to better visualize antioxidant activity, the polysaccharide was tested against commercially available free radical, DPPH. Results indicated that the extract possessed strong antiradical potential as the quenching effect was 15.37, 35.56, and 54.32% at 500, 1000, and 1500 μg/ml concentrations, respectively (**Figure 2C**). Nevertheless, ABTS radical cation, a widely followed superior antioxidant assay (Menghini et al., 2018), was also used herein and the consequence has been depicted in **Figure 2D**. As the concentration ranged from 100, 500, to 1000 μg/ml, inhibition effect amplified from 13.19, 43.98, to 65.78%. Further to evaluate the lipid peroxidation inhibition ability, Rusenan was tested against β-carotene bleaching technique using varying doses. As shown in **Figure 2E**, it readily responded to this antioxidant assay while the activities increased in a concentration-wise pattern. At the level of 100 and 300 μg/ml of extract the bleaching inhibition values were 30.48 and 39.63%, respectively; while application of 500 μg/ml caused more than 50% of inhibition. Moreover, ferricyanide/prussian blue assay was carried out to determine reducing power of Rusenan. According to the results represented in **Figure 2F**, the polysaccharide exhibited moderate reducing power which incremented with the rise of concentration. At level of 2000 and 3000 μg/ml concentration reducing power were 0.27 and 0.39 that gradually elevated to 0.51 at the dose of 4000 μg/ml. Nevertheless, according to the result of total antioxidant activity, reducing capacity of 1 mg of Rusenan was equivalent to 40 ± 1 μg of BHA. Finally, metal ion binding capacity of the fraction was estimated following chelating ability of ferrous ion method. In this assay, a concentration response trend was observed in respect to Fe^2+^ binding ability of the extract (**Figure 2G**). At the concentration of 50, 100, and 200 μg/ml, Rusenan was able to chelate 44.54, 60.92, and 71.51% of ferrous ions, respectively.

**Table 2:** Antioxidant activity of crude polysaccharide, Rusenan, isolated from *Russula senecis*.

	Antioxidant assays	Rusenan	Standard
EC_50_ value (μg/ml)	Scavenging ability of superoxide radicals	360 26a	261 36b
	Scavenging ability of hydroxyl radicals	403 10a	69 1b
	Scavenging ability of DPPH radicals	1387 52a	2.15 0.25b
	Scavenging ability of ABTS radicals	638 13a	3.65 0.02b
	Inhibition of β-carotene bleaching	488 36a	4.53 0.61b
	Chelating ability of ferrous ion	80 2a	2.54 0.5b
	Reducing power	3885 121a	14.5 5b
Total antioxidant activity by phosphomolybdenum method (μg BHA equivalent/mg of dry polysaccharide)		40 1	_


*The results are presented in EC_*50*_ values (mean ± SD; n = 3) corresponding to 50% of antioxidant activity or 0.5 of absorbance for reducing power assay. BHA was used as standard in all assays except chelating ability of ferrous ion method where EDTA was adopted as a positive control. In each row, different letters mean significant differences between sample and standard (p <0.05).*

According to previous literature, the antioxidant potential of natural polysaccharides might be influenced by their architecture like monosaccharide component, water solubility, polarity, molecular weight, structure of chain conformation and intramolecular hydrogen bonds. In this context, some researchers have also reported that polymers with more rhamnose and mannose content are able to display better antioxidant activity (Wang et al., 2017). Thus, it could be assumed that the remarkable antioxidant effect of Rusenan was not due to a single element but the result of combination of many factors. Consequently, comparative study revealed that the fraction from *R. senecis* might possess stronger radical scavenging potential than crude as well as pure polysaccharides extracted from *R. virescens* (Sun et al., 2010). The data also implied that Rusenan exhibited better chelating ability than polysaccharidic extract from *P. ostreatus* (Mitra et al., 2013) and *P. florida* (Saha et al., 2013). Recently, antioxidant activity of partially purified crude polysaccharide extract from the mushroom, *S. commune*, have been reported which appeared to be much poorer than Rusenan (Klaus et al., 2011). Notably, Rusenan was also detected to be more effective than the alkaline extracted polysaccharide from *R. senecis* (Khatua and Acharya, 2017). Overall, it can be conferred that Rusenan owns relatively strong antioxidant ability in contrast to other mushrooms as reflected in all aforementioned assays.

### Determination of Immune-Stimulatory Potential of Rusenan

#### Effect on RAW 264.7 Cell Proliferation and Phagocytic Activity

**FIGURE 3:**
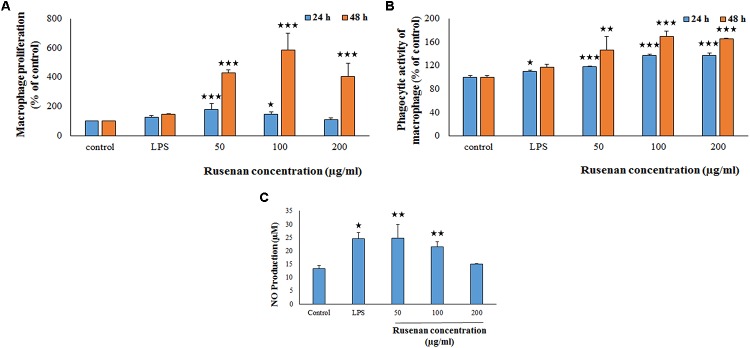
Effect of crude polysaccharide, Rusenan, from *Russula senecis* on macrophages. **(A)** Proliferation was monitored in treatment of the fraction for 24 and 48 h by WST method and expressed in relation (%) to control. **(B)** Phagocytosis in relation (%) to control was determined by neutral red method. **(C)** Release of NO in cell supernatant was quantified using Griess reagent. In all assays LPS at the concentration of 5 μg/ml was used as positive control. Results represent the mean ± SD of at least three independent experiments. ANOVA *p* < 0.05; as regards Tukey’s *post hoc* test the sign ‘^∗^’ indicates significant differences compared to untreated control group. ^∗^*p* < 0.05, ^∗∗^*p* < 0.01, ^∗∗∗^*p* < 0.001.

Macrophages play an important role in host defense that phagocytize the pathogens. In that circumference, if the number of the monocyte is proliferated then it represents a significant indispensable step of immunological defense system (Venter et al., 2014). Thus search for immune-boosting drugs that can potentiate propagation and phagocytic activity rate are one of the corner-stone in modern medicine. Results clearly indicated that Rusenan promoted these phenomena in a dose as well as time dependent manner. As shown in **Figure 3A**, after treatment of the fraction (50, 100, and 200 μg/ml) for 24 h, viability of macrophage cells amplified to 180.06, 148.61, and 108.49%, respectively, in comparison to negative control. While after 48 h the proliferation elevated to 430.33, 584.74, and 405.08% at those tested concentrations. On the other hand, LPS incremented proliferation up to 127.57 and 147.74% after 24 and 48 h treatment, respectively.

The phagocytic activity increased by 117.63, 137.16, and 137.19% after 24 h incubation due to treatment of 50, 100, and 200 μg/ml of Rusenan while after 48 h the activity was 146.41, 169.06, and 165.49% in comparison with control. In contrast to that, LPS induced 109.7 and 116.67% after treatment of 24 and 48 h, respectively (**Figure 3B**). Such observation allowed to conclude that Rusenan presented a safety profile to macrophages over the tested concentrations and definitely possessed an immune-stimulatory effect that was even higher than the positive control.

#### Effect on NO and ROS Production in Macrophages

**FIGURE 4:**
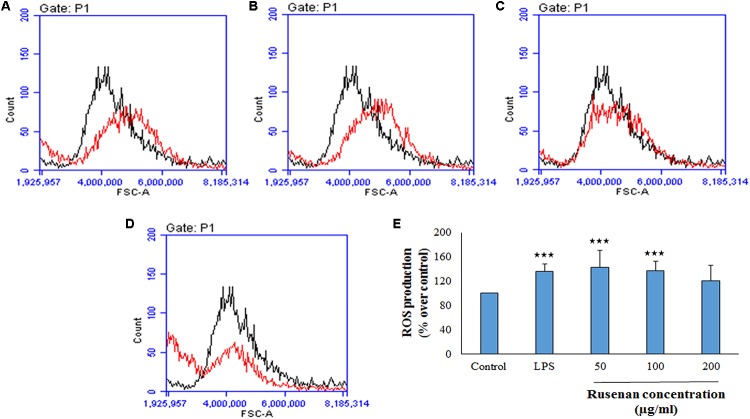
Effect of crude polysaccharide, Rusenan, isolated from *Russula senecis* on intracellular production of ROS in macrophages. RAW 264.7 cells were treated with the fraction or LPS, and after 24 h, intracellular ROS generation was determined by flow cytometry using DCFDA dye. Graphs represent log fluorescence intensity of oxidative product of DCFDA treated with **(A)** LPS at 5 μg/ml concentration and Rusenan at variable doses such as **(B)** 50 μg/ml **(C)** 100 μg/ml **(D)** 200 μg/ml concentration in comparison with negative control. **(E)** Relative fluorescence intensity was also analyzed in detail. Data were represented as mean ± SD of three independent experiments. ANOVA *p* < 0.05; as regards Tukey’s *post hoc* test the sign ‘^∗^’ indicates significant differences compared to untreated control group. ^∗∗∗^*p* < 0.001.

In addition to phagocytosis, production of ROS and NO by macrophages also play a key role in the process of bacterial killing. NO works in combination with superoxides or hydrogen peroxide generating peroxynitrite radicals that can kill engulfed microbes in phagosome. Thus, increase in NO and ROS production by the innate immune cell can be used as a representation of macrophage activation state (Forman and Torres, 2002; Razali et al., 2014). As shown in **Figure 3C**, Rusenan exhibited potential on stimulating NO production in a concentration dependent manner. At the doses of 50, 100, and 200 μg/ml, the fraction induced 24.78, 21.5, and 14.95 μM NO production in RAW 264.7 cells, respectively, while in control and LPS stimulated sets 13.33 and 24.47 μM NO was quantified. Data also showed that Rusenan was capable of inducing intracellular ROS production as the fluorescence intensity increased by 142.5, 137.276, and 120.037% for 50, 100, and 200 μg/ml treated cells, respectively, in comparison to negative control (**Figure 4**). Whereas LPS could enhance the oxidative bursts only by 135.722% in RAW264.7 cells.

#### Detection of Morphological Changes of Macrophages

**FIGURE 5:**
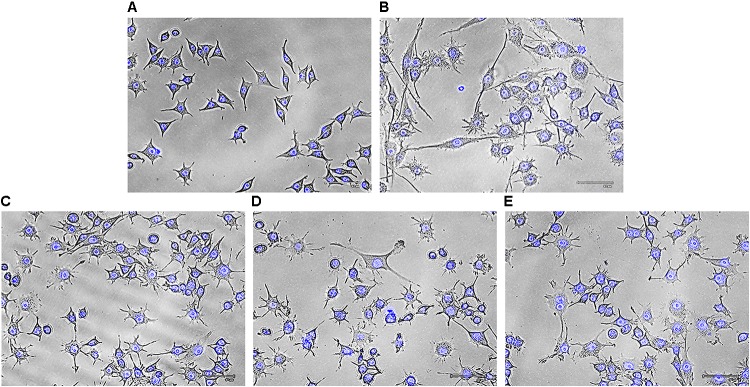
Effect of crude polysaccharide, Rusenan, isolated from *Russula senecis* on morphology of macrophages. Cells were incubated for 24 h with different concentrations of Rusenan where LPS at 5 μg/ml concentration was used as a positive control. Afterwards, cells were fixed, stained with DAPI, subjected to fluorescence microscopy and images were captured. **(A)** Negative control, **(B)** LPS, **(C)** 50 μg/ml, **(D)** 100 μg/ml, **(E)** 200 μg/ml.

To eliminate foreign matter and apoptotic cells, macrophages migrate and continuously survey the environment to detect damaged tissue or invading pathogens. After encountering stimuli, morphology of macrophage cells is altered due to formation of filopodia and lamellipodia from surface edges containing actin bearing spikes and actin filament, respectively. Such morphodynamics is important for the monocyte for attachment to extracellular matrix that will further help to migrate to inflammatory sites (Kim et al., 2015). To investigate effects of Rusenan on macrophage activation, RAW 264.7 cells were incubated with the extract for 24 h. Microscopic analysis showed that LPS as well as the fraction at all investigating doses induced change in cell size from rounded structure to dendritic construction. Besides, the treatments also alleviated production of thin sheets extension from cell boundaries in contrast to unstimulated cells (**Figure 5**).

#### Estimation of Gene Expression by RT-PCR Analysis and Possible Mechanism of Action

**FIGURE 6:**
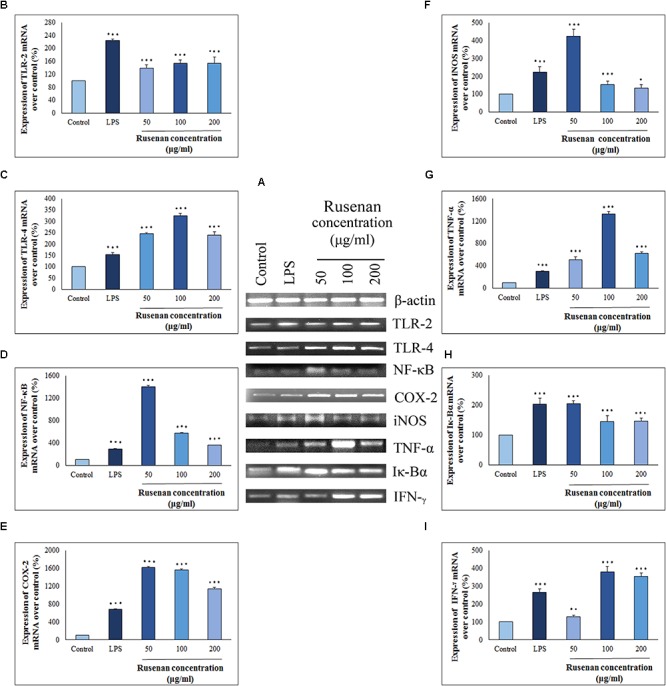
Analysis of mechanism of action by crude polysaccharide, Rusenan, isolated from *Russula senecis* in RAW 264.7 cells. **(A)** Total RNA was isolated from macrophage cells after 24 h incubation either with LPS (5 μg/ml concentration) or Rusenan (50, 100, and 200 μg/ml concentration) along with untreated cells. cDNA was prepared from respective RNA samples, and semi-quantitative reverse transcriptase-PCR was performed to analyze the expression of eight different genes where β-Actin was considered as a house keeping gene. Further, the band intensities were quantified by ImageJ software to signify increase in transcription level of corresponding genes in relation (%) to control: **(B)** TLR-2, **(C)** TLR-4, **(D)** NF-κB, **(E)** COX-2, **(F)** iNOS, **(G)** TNF-α, **(H)** Iκ-Bα, **(I)** IFN-γ. Values were represented as mean ± SD of at least two independent experiments. ANOVA *p* < 0.05; as regards Tukey’s *post hoc* test the sign “^∗^” indicates significant differences compared to untreated control group. ^∗^*p* < 0.05, ^∗∗^*p* < 0.01, ^∗∗∗^*p* < 0.001.

Cytokines and chemokines are potent signaling molecules produced by macrophages which link innate and adaptive immunity. However, secretion of them requires activation of TLRs and NF-κB pathways that regulate a number of genes (Sun et al., 2017). In order to investigate whether these mediators play a role in Rusenan mediated stimulation of macrophages, the crude polysaccharide was incubated with RAW 264.7 cells for 24 h. As presented in **Figure 6**, the levels of all investigating genes were visibly increased in treatment of 50, 100, and 200 μg/ml of the fraction in comparison to negative control. Analysis suggested that Rusenan at 50 μg/ml concentration was the most potent to induce transcriptional activation of mediators and cytokines. At that concentration, the mRNA levels of TLR-2, TLR-4, NF-κB, COX-2, iNOS, TNF-α, Iκ-Bα, and IFN-γ were significantly improved by 138.7, 245.7, 1406.5, 1615, 423.01, 511.7, 205.6, and 127.6%, respectively. Conversely, LPS upregulated these genes only by 224.1, 153, 293.3, 680.1, 222.69, 105.7, 204.2, and 265.1% indicating strong promoting effect of Rusenan.

To establish the immune-boosting effect of Rusenan it is important to elucidate molecular mechanism of action. The fraction being composed of different monosaccharide moiety might not be able to penetrate macrophage cell membrane. Rather they might interact with pattern recognition receptor like TLR2/4 on surface of macrophages as they recognize β-glucan. As a result, downstream signaling pathway was triggered that in turn activated NF-κB, the most important regulator of gene expression in monocyte. Stimulation of the transcription factor was reflected by expression of a number of inflammatory chemokines or cytokines. Besides, activated NF-κB attached to the promoter region of its own as well as inhibitor gene as it autoregulates its synthesis. Eventually, TNF-α, IFN-γ and COX-2 were secreted in response to Rusenan. In addition, macrophages also expressed iNOS that promoted metabolism of arginine into NO fostering a highly microbicidal environment.

## Conclusion

Overall, Rusenan with glucose (mainly β-glucan) as major component could be regarded as a potent free radical scavenger and murine macrophage stimulator. In the view of antioxidant activity, the crude polysaccharide exhibited high potential in scavenging superoxide radicals, inhibition of OH^.^ generation, stabilizing DPPH^.^, quenching ABTS radical, inhibition of β-carotene bleaching, reducing power and chelating ability of Fe^2+^ as revealed by low EC_50_ values. Besides, the sample also exhibited strong immune-stimulation activities in which at only 50 μg/ml concentration it may initiate innate immunity by promoting macrophage proliferation, phagocytosis, morphological changes, NO release, ROS production, transcription of TLR-4, TLR-2, NF-κB, COX-2, iNOS, TNF-α, IκB-α, and IFN-γ. Considering outcomes in the present study, it can be confirmed that Rusenan can be developed individually as a powerful biological response modifier and standard antioxidant drug.

## Author Contributions

SK carried out all the experiments presented in this manuscript. KA conceived and designed the experiments. SK wrote and constructed the manuscript.

## Conflict of Interest Statement

The authors declare that the research was conducted in the absence of any commercial or financial relationships that could be construed as a potential conflict of interest.
